# A BERT-Based Aspect-Level Sentiment Analysis Algorithm for Cross-Domain Text

**DOI:** 10.1155/2022/8726621

**Published:** 2022-06-27

**Authors:** Ning Liu, Jianhua Zhao

**Affiliations:** ^1^College of Economics Management, Shangluo University, Shangluo 726000, China; ^2^Engineering Research Center of Qinling Health Welfare Big Data, Universities of Shaanxi Province, Shangluo 726000, China; ^3^College of Mathematics and Computer Application, Shangluo University, Shangluo 726000, China

## Abstract

Cross-domain text sentiment analysis is a text sentiment classification task that uses the existing source domain annotation data to assist the target domain, which can not only reduce the workload of new domain data annotation, but also significantly improve the utilization of source domain annotation resources. In order to effectively achieve the performance of cross-domain text sentiment classification, this paper proposes a BERT-based aspect-level sentiment analysis algorithm for cross-domain text to achieve fine-grained sentiment analysis of cross-domain text. First, the algorithm uses the BERT structure to extract sentence-level and aspect-level representation vectors, extracts local features through an improved convolutional neural network, and combines aspect-level corpus and sentence-level corpus to form a sequence sentence pair. Then, the algorithm uses domain adversarial neural network to make the feature representation extracted from different domains as indistinguishable as possible, that is, the features extracted from the source domain and the target domain have more similarity. Finally, by training the sentiment classifier on the source domain dataset with sentiment labels, it is expected that the classifier can achieve a good sentiment classification effect in both source and target domain, and achieve sentence-level and aspect-level sentiment classification. At the same time, the error pooled values of the sentiment classifier and the domain adversary are passed backwards to realize the update and optimization of the model parameters, thereby training a model with cross-domain analysis capability. Experiments are carried out on the Amazon product review dataset, and accuracy and F1 value are used as evaluation indicators. Compared with other classical algorithms, the experimental results show that the proposed algorithm has better performance.

## 1. Introduction

With the vigorous development of social media such as online comments, Weibo, WeChat, and forum communities, a large amount of subjective text data with emotion is presented on the Internet [1]. Emotion specifically refers to emotional polarity (tendency), which is a concrete manifestation of individual users' emotional cognition and evaluation of products, services, or social public opinion [2, 3]. Subjective texts in social media contain rich emotional information. By mining the emotional category information of these texts, it can provide technical support for applications such as product recommendation, customer management, word-of-mouth analysis, news review analysis, and stock recommendation, which is extremely important research value [3].

Sentiment analysis is a very domain-dependent task. The sentiment characteristics of data in different domains are not exactly the same. The sentiment prediction model trained on data in a certain domain cannot usually be used directly in other domains [4]. In the face of new domain, in order to build a good sentiment prediction model, it is necessary to manually label the data. However, high-quality human-labeled data is an expensive process. At the same time, existing research has accumulated labeled sentiment data in some domain, and it is a pity to completely discard these data. As one of the important problems in natural language processing tasks, cross-domain text sentiment classification has always been a research hotspot and difficulty in industry and academia [5].

Transfer learning uses labeled training samples in the source domain to build a reliable model to predict unlabeled samples in the target domain with different data distributions [6]. A large number of existing research works show that transfer learning is one of the effective means to solve cross-domain text sentiment classification. Some researchers have carried out preliminary research on this issue and achieved some results.

Robert et al. [7] proposed a domain adaptation method to select the most similar samples from the source domain training set to the target domain, and evaluated this method in semisupervised cross-domain text-level sentiment classification experiments. Xia et al. [8] proposed a joint transfer strategy based on feature ensemble and sample ensemble. They first used a feature ensemble strategy to learn a new labeling function to recompute new features, used PCA-based feature selection for instance adaptation, and achieved an average accuracy of 77.5% on Amazon's 4 product review datasets. Tareq et al. [9] used the conditional probability joint association to measure the transfer characteristics of the source domain and the target domain, and applied the naive Bayes model and three feature selection methods (information entropy, odds ratio, and chi-square test) to cross-domain sentiment classification task. Yu et al. [10] used a neural network architecture to study the problem of cross-domain sentiment classification, using two auxiliary tasks to improve the performance of sentence embedding on cross-domain sentiment classification, achieving 79.6% average accuracy on the English movie, camera, laptop, and restaurant datasets.

In the cross-domain sentiment classification task of text-based social media, it is necessary to deeply understand the mechanism of language expression and the mechanism of emotion transfer. It is very difficult to build large-scale, high-quality labeled datasets; however, deep transfer learning can significantly reduce the demand for labeled data in the target domain. Therefore, deep transfer learning is widely used in cross-domain sentiment classification tasks, and has achieved good results.

Zhang et al. [11] proposed an interactive attention transfer network (IATN) for cross-domain text sentiment classification tasks. IATN provides an interactive attention transfer mechanism that can better transfer emotion by integrating sentence and aspect information. Ji et al. [12] designed a bifurcated-LSTM network utilizing attention-based LSTMs, augmented datasets, and orthogonal constraints. This method can extract domain-invariant sentiment features from source domains and perform sentiment analysis in different target domains. The method achieves an average accuracy of 80.92% for cross-domain sentiment classification on Amazon's 7-domain data. Zhang et al. [13] proposed hierarchical attention generative adversarial networks (HAGAN for short). It generates a document representation by alternately training a generator (generative model) and a discriminator (discriminative model), which is sentimentally distinguishable but domain-indistinguishable, and achieves an average accuracy of 81.56% on Amazon 4 review datasets. Liu et al. [14] proposed a fuzziness-based domain-adversarial neural network with autoencoder (Fuzzy-DAAE for short). Omar et al. [15] introduced text generation in the target domain as a labeled dataset in the target domain, and compared text generation based on deep learning such as LSTM, RNN. and Markov chain-based text generation, the accuracy rate of 72.0% is achieved on Kitchen as the target domain dataset. Cai et al. [16] used a denoising autoencoder to extract deeper shared features with robustness, and used a combination of Wasserstein distance-based domain adversarial and orthogonal constraints to better extract deep shared features across different domains for cross-domain text sentiment classification task.

However, although the existing work has achieved great success by introducing transfer learning or domain adaptation mechanism to solve cross-domain text sentiment classification tasks, the research on many important issues is not perfect and in-depth, and there are still many theoretical and technical problems need to be explored [17–20]. For example, the traditional cross-domain text sentiment classification is often to achieve text-level sentiment transfer between different domains, and less attention is paid to the cross-domain fine-grained sentence-level, aspect-level, and attribute sentiment orientation task research. For example, in the evaluation text, “This restaurant is so delicious, but the attitude of the waiter is too cold”. The emotional polarity for “taste” is positive, and the emotional polarity for “service” is negative, the sentiment polarity is neutral for the “environment” aspect, and this type of problem requires fine-grained sentiment analysis from the aspect level. At the same time, in the existing research, only the word-level features are considered when extracting shared sentiment features, and the language features of the text, such as the semantic information contained in the context, are not considered. When the sentence lacks emotional words or expresses irony, it is difficult to perform accurate sentiment classification if the semantics and other related information of the sentence are ignored [21–23].

In order to effectively achieve the performance of cross-domain text sentiment classification, this paper proposes a BERT-based aspect-level sentiment analysis algorithm for cross-domain text to achieve fine-grained sentiment analysis of cross-domain text. First, the algorithm uses the BERT structure to extract sentence-level and aspect-level representation vectors, extracts local features through an improved convolutional neural network, and combines aspect-level corpus and sentence-level corpus to form a sequence sentence pair. Then, the algorithm uses domain adversarial neural network to make the feature representation extracted from different domains as indistinguishable as possible, that is, the features extracted from the source domain and the target domain have more similarity. Finally, by training the sentiment classifier on the source domain dataset with sentiment labels, it is expected that the classifier can achieve a good sentiment classification effect in both source and target domain, and achieve sentence-level and aspect-level sentiment classification. At the same time, the error pooled values of the sentiment classifier and the domain adversary are passed backwards to realize the update and optimization of the model parameters, thereby training a model with cross-domain analysis capability. Experiments are carried out on the Amazon product review dataset; accuracy and F1 value are used as evaluation indicators. Compared with some existing classical algorithms, the results show that our proposed algorithm has better performance.

The organization of this paper is as follows. In Section 2, a review of current literature is provided. In Section 3, the detailed description of the proposed method is presented. In Section 4, the experimental results and analysis are provided. Finally, a short summary is included in Section 5.

## 2. Related Works

### 2.1. BERT

BERT is modeled through a self-attention mechanism, which can directly obtain the global information of the text. Since it has no forgetting gate mechanism, the information of all words is preserved, so BERT can better express the complete semantic information of the sentence, and can also directly find the correlation features between words from the global word features.

The BERT model is composed of multiple transformer layers and uses a multihead attention mechanism. After the input vector is multilayered linearly transformed to obtain different linear values, it is then input to the attention module to calculate the attention weight. The output value of the attention mechanism is combined with the previous linear change again, and the final output of the multihead attention mechanism can be obtained. For any vector input to Transformer, it is processed and output, Trans(.) represents all operations in Transformer, as shown in the following formula:

(1) $$\begin{array}{c} V_{t}=\text{Trans}\left(W_{t}X_{a}+b_{t}\right), \end{array}$$

where *V*_*t*_ represents the output vector of the Transformer and *X*_*a*_ represents the input vector.

BERT is formed by stacking multiple Transformers together, and Bert(.) represents the calculation process in Bert, as shown in the following formula:

(2) $$\begin{array}{c} V_{b}=\text{Bert}\left(W_{b}X_{b}+b_{b}\right), \end{array}$$

where *V*_*b*_ represents the output value of BERT and *X*_*b*_ represents the input vector.

### 2.2. Aspect-Level Sentiment Analysis

Aspect-level sentiment analysis is a fine-grained sentiment classification task in sentiment analysis, whose purpose is to identify the sentiment polarity expressed by a sentence on an aspect. There are usually two methods for aspect-level sentiment analysis: (1) Traditional machine learning methods are used, relying on artificially constructed features and rules, but such methods are very time-consuming and labor-intensive. (2) The deep learning method is used to introduce the neural network into the research field of sentiment classification, which can automatically select the features without manual intervention, greatly alleviate the model's dependence on feature engineering, and enable the model to achieve better performance at a lower cost. For example, literature [24] uses pretrained word vectors to apply CNN to text classification tasks. Literature [25] uses the LSTM network to model the text sequence semantically, and the sentence expression modeled by the LSTM can reflect the semantic connection of the text context. However, neural network-based methods cannot effectively distinguish the importance of each word in a sentence, and the sentiment polarity of a sentence is not only determined by the content, but also closely related to the aspects involved. For this reason, some scholars introduce attention mechanisms to focus on important information in sentences. For example, Reference [25] proposed two different attention-based bidirectional long-short-term memory network models for target-related sentiment classification. Reference [26] used an attention-based deep memory network for aspect-based sentiment analysis tasks.

Research shows that the above methods only encode text semantic information using word embedding technology, ignoring syntactic structure information and word frequency information, which play an important role in preserving structural information and help shorten the distance between aspect words and opinion words [27–30]. If the dependencies on the syntactic path cannot be used correctly, the function of syntactic structure cannot be fully exerted. Recently, some scholars have used graph-based models to integrate syntactic structures. Sun et al. [31] transformed the dependency tree into a graph and learned the GCN on the dependency tree to model the structure of sentences, propagating information from syntactic neighborhood opinion words to aspect words.

### 2.3. Transfer Learning

Transfer Learning (TL) is a technique that allows fine-tuning of existing model algorithms to apply to new domains or functions [32]. In transfer learning, researchers usually divide data into source data and target data. The purpose of transfer learning is to apply general knowledge to new related tasks under the premise of acquiring some additional data or existing models to make full use of the source data to help the model improve its performance on the target data. According to the relationship between the source domain and the target domain, transfer learning methods can be divided into three categories [33]: instance-based transfer learning, model parameter-based transfer learning, and feature-based transfer learning. Among them, instance-based transfer learning is a relatively simple transfer learning method. This method selects examples from the source domain that is useful for training in the target domain and is used as a supplement to the training set to expand the training set of the target domain, thereby improving the migration effect [34]. The main idea of model parameter-based transfer learning is to make the source domain and target domain share model parameters [35]. That is, the neural network model is pretrained in the source domain through a large amount of source domain data, and then the pretrained model is directly applied to the target task. In this process, all or part of the model parameters can be reused. Feature-based transfer learning is divided into feature extraction-based transfer method and feature-mapping-based transfer method. Feature extraction-based transfer method reuses pretrained local networks in the source domain and turns them into part of a deep network in the target domain; feature-mapping-based transfer method maps instances from source and target domains to new data space. In the new data space, the instances from the two domains have similar data distribution, which is suitable for joint deep neural network. The advantage is that by adjusting the data distribution, the training set can be increased, thereby improving the transfer effect.

## 3. Our Method

### 3.1. Basic Definition

Cross-domain text sentiment analysis refers to using only sentiment-labeled data in the source domain to train a sentiment classifier and use it for sentiment classification on the target domain data. Given a source domain dataset *D*_*s*_={(*x*_*s*_^*i*^, *a*_*s*_^*i*^), *y*_*s*_^*i*^}_*i*=1_^*N*_*s*_^, target domain dataset *D*_*t*_={(*x*_*t*_^*j*^, *a*_*t*_^*j*^)}_*j*=1_^*N*_*t*_^, where *x* represents a sentence, a represents an aspect word in sentence *x*, *y*_*s*_ represents the sentiment label corresponding to aspect word *a*, *Ns* represents the amount of data with sentiment labels in the source domain, and *N*_*t*_ represents the amount of data without sentiment labels in the target domain. Cross-domain tasks need to learn a sentiment classifier on *D*_*s*_ to achieve sentiment polarity classification for *D*_*t*_.

### 3.2. Algorithmic Framework

**Figure 1:**
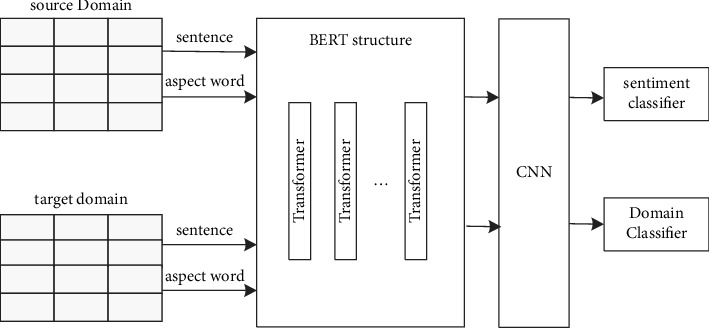
Aspect-level sentiment analysis model.

Based on the ideas of BERT model, convolution model, and adversarial model, the model structure proposed in this paper is shown in Figure 1. In the model, the input data is a matrix of sentence word and aspect word representations in the source and target domain texts. Feature extraction consists of BERT and CNN sharing weights. First, the feature representation covering the semantic information of the sentence is extracted by the BERT model; then the CNN is combined to further extract the key local features in the feature representation. At the same time, the features with a large amount of sentence semantic information are further reduced in dimension; finally, the output feature of the CNN is used as the domain inputs to adversarial classifiers and sentiment classifiers. The domain adversarial classifier is used to achieve domain confusion, and the sentiment classifier is used to achieve aspect-level sentiment classification of the data.

**Figure 2:**
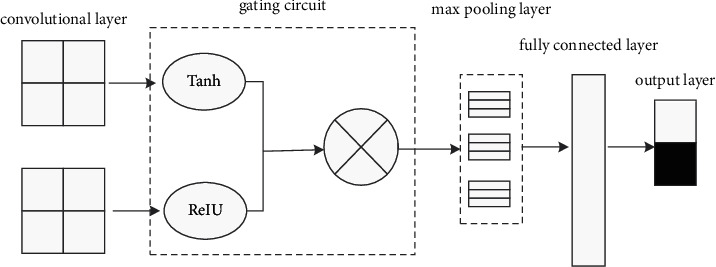
Improved CNN structure.

In order to improve the performance of the CNN in Figure 1, we have modified the structure of the CNN, and the modified results are shown in Figure 2. As shown in Figure 2, we have improved the CNN and added a gated activation unit in CNN. When the aspect information and emotional information pass through the activation unit, the model will give emotional words with closer aspect information high weights to improve the classification accuracy of aspect-level sentiment analysis. On the contrary, if the relationship between the two is far away, the weight given to the emotional word may be very small or 0.

During model training, the data in the source domain is extracted into the sentiment classifier after feature extraction, while the data in the target domain is combined with the features extracted in the source domain after extraction, and then used as the input of the domain adversarial classifier. The error pooled values of the sentiment classifier and the domain adversarial classifier are back-passed to enable the updating and optimization of model parameters, thereby training a model with cross-domain analysis capabilities.

### 3.3. Implementation Process

#### 3.3.1. BERT

BERT is modeled through a self-attention mechanism, which can directly obtain the global information of the text. BERT can better express the complete semantic information of the sentence, and can also directly find the correlation features between words from the global word features.

In general, BERT is applied to sentence-level sentiment classification, which is defined as a single-sentence classification task. However, in aspect-level sentiment analysis, the same sentence can express different views on different aspects, and express different sentiments. Traditional sentence-level sentiment classification is limited. For example, in the evaluation text, “This restaurant is so delicious, but the attitude of the waiter is too cold”, the emotional polarity for “taste” is positive, and the emotional polarity for “service” is negative, while the emotional polarity for the “environment” aspect is neutral.

To address this issue, this paper considers aspect-level sentiment classification as a sentence pair classification task. In text representation, the special token “[CLS]” (classification) is placed at the beginning of the sequence, and the special token [SEP] is placed in front of the sentence. A sequence sentence pair is formed by the combination of aspect-level corpus and sentence-level corpus, which is vectorized as the input value of BERT. The basic idea is as follows:

First, suppose that an aspect is represented as {*w*_1_, *w*_2_,…, *w*_*N*_} and a sentence is represented as {*w*_1_^`^, *w*_2_^`^,…, *w*_*N*_^`^}. The input sequence is combined using aspects and sentences, and the special token “[CLS]”is placed at the beginning of the sequence, and the special token [SEP] is placed in front of the sentence, forming a sequence of sentence pairs. The expression method is shown in the following formula:

(3) $$\begin{array}{c} I_{A}=\left\{\left[\text{CLS}\right],w_{1},w_{2},\ldots ,w_{N},\left[\text{SEP}\right],w_{1}^{\prime },w_{2}^{\prime },\ldots w_{N}^{!},\left[\text{SEP}\right]\right\}. \end{array}$$

Then, the input sequence is encoded with BERT, and the output vector corresponding to “[CLS]” is represented as an aspect-level sentence. The use of BERT is shown in the following formula:

(4) $$\begin{array}{c} T_{\left[\text{CLS}\right]}=\text{BERT}\left(I_{A}\right). \end{array}$$

Finally, the input of aspect-level sentence representation is performed by a classifier consisting of convolutional layers and Softmax layers for sentiment classification.

#### 3.3.2. CNN Text Convolution Process

After obtaining the output value of BERT, the text convolution structure is used to convolve the output value of BERT, which can not only extract better local text features, but also reduce the dimension of shared emotional features. For each BERT output vector T[CLS] input to the convolutional neural network, the modified CNN is used for convolution processing. During the convolution process, convolution kernels of different sizes are selected to obtain the output value *R* of the convolution, then the results are merged together to form the final feature after max pooling. The use of convolution is shown in the following formula:

(5) $$\begin{array}{c} R=\text{Conv}\left(W_{T}T_{\left[CLS\right]}+b_{T}\right). \end{array}$$

#### 3.3.3. Sentiment Classifier

If only the domain classifier exists, there is no guarantee that the information extracted by the feature extraction module is valid. In order to ensure that the information extracted by the feature extraction module can be used for classification, it is also necessary to rely on a category classifier, which is a sentiment classifier in the current task. The classification accuracy is ensured by supervised training using the sentiment-labeled data in the source domain.

The sentiment classifier is only for the source domain dataset, and the text representation of the source domain is the value obtained after convolution pooling, which is input to Softmax for predicting sentiment classification. The classification is performed as shown in the following formula:

(6) $$\begin{array}{c} y=\text{Softmax}\left(W_{R}R+B_{R}\right). \end{array}$$

#### 3.3.4. Domain Confrontation

Domain confrontation is to generalize the feature properties of the source domain to the target domain, so that the classifier cannot distinguish whether the feature is from the source domain or the target domain, so as to realize the confusion of domain features. In this paper, after the features are extracted from the source domain and the target domain through the feature extractor, while training the source domain sentiment classifier, the features extracted from different domains are input together into the domain classifier for domain classification. Logistic regression is used to build a domain classifier as a domain adversarial structure, and the domain classifier cannot distinguish whether the features are from the source domain or the target domain, so as to achieve the effect of domain confrontation and make the extracted shared sentiment features similar. Assuming that the feature vector of the source domain text after passing through BERT-CNN is HS, and the feature vector of the target domain text passing through BERT-CNN is HT, the two are combined according to formula (7). Then the gradient reversal layer GRL (Gradient Reverse Layer) runs on HD, and the predicted domain category label is shown in the following formula:

(7) $$\begin{array}{c} H^{D}=H^{S}⊕H^{T}, \end{array}$$

(8) $$\begin{array}{c} {\hat{y}}^{d}=\text{soft }\max\left(W_{d}\text{ GRL}\left(H^{D}\right)+b_{d}\right). \end{array}$$

#### 3.3.5. Objective Function

During the training process of the overall model, the two loss functions are merged together to form the final objective function of the model. One loss function is the training objective function of sentiment classifier, used for sentiment classification; one loss function is the objective function used for domain adversarial training, which is used to achieve domain adaptation. The labels of the sentiment classifier can only come from the source domain, while the label information of the domain confrontation is a mixture of the source domain and target domain labels. All parameters are updated through the back-propagation algorithm. The loss function is shown in the following formula:

(9) $$\begin{array}{c} \min L=\sum_{d=1}^{N}L_{\text{senti}}^{d}+\beta L_{\text{domain}}. \end{array}$$

Here, *L* represents the loss function, *d* represents the number of domains, *L*_domain_ represents the loss function of the sentiment classifier, *β* is used to control the magnitude of the error provided by the domain adversarial, and *L*_domain_ represents the objective function of domain adversarial training.

## 4. Experiment and Result Analysis

### 4.1. Experimental Data Set

The public data set is the Amazon product review dataset provided by Li et al. [36], which contains reviews of specific products in 5 different fields, such as Books, DVD disk, Electronics, Kitchen appliances, and Videos. The data for each of these domains contains 6000 tagged reviews (3000 positive reviews and 3000 negative reviews), in addition to multiple reviews with no sentiment polarity tags. Detailed statistics for each domain in the datasets are shown in Table 1.

Since the existing sentiment analysis corpus cannot fully meet the needs of this research, we manually annotated the selected Amazon product review dataset to create a data set suitable for cross-domain aspect-level sentiment analysis tasks. The specific method is to analyze the aspect information and sentiment information on the basis of the sentence-level sentiment analysis public data set, extract the aspect words, and mark the sentiment expressed in the sentence for the aspect. In order to avoid the problems of insufficient training data, different distributions or imbalanced data categories affecting the performance of the model, the corpus created in this research has been manually screened, and the amount of data in each domain and the number of positive and negative labels are basically balanced.

In the experiment, one data set in five different fields is used as the source domain data set, and the other four datasets are used as the target domain data set. The data is divided into training set and test set. The source domain and target domain are trained with 2000 positive texts and 2000 negative texts, respectively. All 6000 pieces of target domain data are used for target domain sentiment polarity prediction during testing.

In this experiment, Bert-base is set as the basic model, and the learning rate is set as 2e-5, which will be used to fine tune and emotion classification process. The development environment is Python 3.6 and tensorflow 1.12.0.

### 4.2. Evaluating Indicator

In the experiment of this paper, the accuracy rate Acc and F1 value are used as evaluation indicators. The accuracy rate represents the ratio of the number of samples correctly classified by the classifier to the total number of samples for a given test data set. The F1 value is a concept proposed on the basis of Precision and Recall to evaluate Precision and Recall as a whole. The F1 value is the harmonic mean of precision and recall.

The calculation of the accuracy rate Acc is shown in the following formula:

(10) $$\begin{array}{c} \text{Acc}=\frac{\sum_{i=1}^{N}\left|y_{i}={\hat{y}}_{i}\right|}{N}, \end{array}$$

where ${\hat{y}}_{i}$ is the predicted label of the data sample, *y*_*i*_ is the actual label of the data sample, and N is the size of the test set.

The evaluation index of data is generally based on the confusion matrix shown in Table 2. The description of TP, TN, FN, and FP is shown below.

- True Positive (TP): It is judged to be a positive sample, and in fact it is a positive sample.
- True Negative (TN): It is judged to be a negative sample and in fact it is a negative sample.
- False Negative (FN): It is judged to be a negative sample, but in fact it is a positive sample.
- False Positive (FP): It is judged to be a positive sample, but in fact it is a negative sample.

Precision represents the proportion of true cases among the predicted positive cases (true cases + false positive cases). The calculation method is shown in the following formula:

(11) $$\begin{array}{c} \text{Precision}=\frac{\text{TP}}{\text{TP}+\text{FP}}. \end{array}$$

The recall rate represents the proportion of true examples in all actual positive examples (true examples + false negative examples). The calculation method is shown in the following formula:

(12) $$\begin{array}{c} \text{Recall}=\frac{\text{TP}}{\text{TP}+\text{FN}}. \end{array}$$

The F1 value is represented by the harmonic average of the precision rate and the recall rate, which is a comprehensive reflection of the precision rate and the recall rate. The calculation method is shown in the following formula:

(13) $$\begin{array}{c} F1_{\text{Score}}=2*\frac{\text{Recall}*\text{Precision}}{\text{Recall}+\text{Precision}}. \end{array}$$

### 4.3. Experimental and Results Analysis

#### 4.3.1. Ablation Experiment

Ablation study refers to understanding the effect of a component on the entire system by studying the performance of an AI system after removing a component. Ablation study requires the system to exhibit graceful degradation: even if a component is lost or weakened, the system can continue to operate while maintaining functionality.

To examine the superiority of aspect-level cross-domain sentiment analysis methods, we conduct two types of ablation experiments on our method. Among them, Experiment 1 explores the advantages of aspect-level sentiment analysis results preprocessed by BERT compared to sentence-level sentiment analysis without BERT preprocessing; Experiment 2 explores the advantages of aspect-level sentiment analysis with gated activation unit compared to sentence-level sentiment analysis without gated activation unit.

The results of Experiment 1 are shown in Table 3. Here, “source” represents source domain and “target” represents target domain. “B” represents “Books”, “D” represents DVD disk, “E” represents Electronics, “K” represents Kitchen appliances, and “V” represents Videos. “NO” indicates the accuracy and F1 value of the model without BERT preprocessing. “YES” indicates the accuracy and F1 value of our model with BERT preprocessing. It can be seen that after preprocessing with BERT, the aspect-level cross-domain sentiment analysis results are better than the aspect-level sentiment analysis without BERT preprocessing in most experiments. The reason is that BERT can mine the semantic information of sentences. For different aspects, the emotional information has a stronger pertinence, which is more conducive to aspect-level sentiment classification. Therefore, it can be concluded that BERT can help the model to better understand sentence semantics, thereby improving the classification accuracy.

The results of Experiment 2 are shown in Table 4. “No” indicates the corresponding accuracy and F1 value of the model without gated activation unit, and “YES” indicates the classification accuracy and F1 value of the model proposed in this paper (with gated activation unit). It can be seen that aspect-level sentiment analysis results with gated units are superior to sentence-level sentiment analysis without gating. The reason is that in the classification algorithm with gated activation unit, the gating unit will select the emotional feature according to the aspect information, weight, which is beneficial to get better classification results. However, when the gating unit is turned off, the correlation between emotional features and aspect information is not fully expressed. Since there is no weighting based on features, it is not conducive to the classification of sentences with complex emotions, and the classification accuracy is low.

#### 4.3.2. Comparing Experiments with Other Methods

In the experimental dataset, the method proposed in this paper is compared with the experimental results of the following methods.

  SCL-ML [37]: The method first uses the interaction information to construct the pivot feature, and then calculates the correlation between the pivot feature and the nonpivot feature of the source domain and the target domain, respectively.
  ITIAD [38]: The method processes the common features of the source domain and the target domain, and applies these features to perform cross-domain sentiment classification.
  CGRU [39]: This method is a combination of Convolutional Neural Network (CNN) and Gated Recurrent Unit (GRU), utilizing the local features generated by CNN and the long-term dependency learned by GRU.

Table 5 shows the accuracy comparison results of our method and other methods under the experimental data set. According to the data in Table 5, it can be seen that the aspect-level cross-domain sentiment analysis method proposed in this paper achieves the best results, surpassing several other classic cross-domain sentiment classification models. Compared with the SCL-ML, ITIAD, and CGRU methods, the average accuracy of our method is improved by 6.4%, 4.1%, and 2.0%, respectively [40].

Table 6 shows the F1 value comparison results of our method and other methods under the experimental data set. According to the data in the table, it can be seen that the aspect-level cross-domain sentiment analysis method proposed in this paper achieves the best results, surpassing several other classic cross-domain sentiment classification models. Compared with the SCL-ML, ITIAD, and CGRU methods, the average F1 value of our method is improved by 5.7%, 3.6%, and 1.9%, respectively.

It shows that the model proposed in this paper can better extract the features of text compared with these classic methods. This is because: (1) the model in this paper uses Bert for preprocessing, which can better express the complete semantic information of sentences, and also directly find the correlation features between words from the global word features. (2) The model in this paper improves CNN by adding a gated activation unit, which can improve the weight of emotional words closely related to aspect information and help to improve the accuracy.

On the one hand, it verifies the feasibility of fine-grained cross-domain sentiment analysis, and on the other hand, it also verifies the advanced nature of the algorithm in this paper. The problem that it is difficult to obtain good classification results due to less labeled data in the target domain is improved, and the model can perform well in many fields.

## 5. Conclusion

In this paper, a BERT-based aspect-level sentiment analysis algorithm for cross-domain text is proposed to achieve fine-grained sentiment analysis of cross-domain text. The BERT structure is used to extract sentence-level and aspect-level representation vectors, an improved convolutional neural network is used to extract local features, and domain adversarial neural network is used to make the feature representation extracted from different domains as indistinguishable as possible. The experimental results show that the proposed algorithm has good performance. In many current application scenarios, the pretrained model contains a lot of knowledge, and it is an interesting direction to build an emotional knowledge graph through the pretrained model. Most of the current work focuses on how to build a knowledge graph model, but few researchers focus on building a knowledge graph for sentiment analysis tasks. The following work will focus on the research of sentiment analysis based on knowledge graph.

## Figures and Tables

**Table 1:** Statistical information of datasets.

Domain	Positive comments	Negative comments	Unmarked comments
Book	3000	3000	9750
DVD	3000	3000	11843
Electronics	3000	3000	17009
Kitchen	3000	3000	13856
Videos	3000	3000	30180

**Table 2:** Confusion matrix.

Category	Actual positive class	Actual negative class
Experimental positive class	TP	FN
Experimental negative class	FP	TN

**Table 3:** Results of accuracy and F1.

Source	Target	Accuracy		F1 value	
		NO	YES	NO	YES
B	D	0.778	0.822	0.772	0.814
	E	0.753	0.785	0.762	0.796
	K	0.768	0.821	0.774	0.835
	V	0.815	0.823	0.809	0.812
	B	0.755	0.835	0.752	0.842

D	E	0.753	0.812	0.760	0.811
	K	0.784	0.814	0.764	0.825
	V	0.878	0.917	0.910	0.921
	B	0.752	0.801	0.750	0.821
	D	0.754	0.801	0.734	0.824

E	K	0.815	0.875	0.812	0.867
	V	0.756	0.792	0.784	0.815
	B	0.712	0.784	0.701	0.765
	D	0.754	0.805	0.762	0.801
	E	0.768	0.822	0.732	0.821
	V	0.815	0.845	0.810	0.848
	B	0.855	0.875	0.840	0.867
	D	0.835	0.869	0.857	0.869
	E	0.824	0.854	0.834	0.868
	K	0.850	0.889	0.846	0.870

**Table 4:** Results of Accuracy and F1 value.

Source	Target	Accuracy		F1 value	
		NO	YES	NO	YES
B	D	0.783	0.822	0.785	0.814
	E	0.767	0.785	0.770	0.796
	K	0.782	0.821	0.790	0.835
	V	0.810	0.823	0.810	0.812
	B	0.776	0.835	0.781	0.842

D	E	0.765	0.812	0.770	0.811
	K	0.794	0.814	0.785	0.825
	V	0.884	0.917	0.901	0.921
	B	0.763	0.801	0.759	0.821
	D	0.765	0.801	0.769	0.824
	K	0.824	0.875	0.835	0.867

E	V	0.767	0.792	0,778	0.815
	B	0.747	0.784	0.746	0.765
	D	0.766	0.805	0.765	0.801

K	E	0.787	0.822	0.779	0.821
	V	0.830	0.845	0.828	0.848
	B	0.862	0.875	0.865	0.867
	D	0.840	0.869	0.852	0.869

V	E	0.831	0.54	0.838	0.868
	K	0.847	0.889	0.864	0.870

**Table 5:** Accuracy of different methods.

Source	Target	Method			
		SCL-ML	ITIAD	CGRU	Ours
B	D	0.734	0.798	0.815	0.822
	E	0.723	0.782	0.778	0.785
	K	0.762	0.805	0.797	0.821
	V	0.752	0.764	0.785	0.823
	B	0.792	0.814	0.825	0.835

D	E	0.771	0.787	0.805	0.812
	K	0.765	0.786	0.796	0.814
	V	0.806	0.835	0.855	0.917
	B	0.784	0.794	0.797	0.801
	D	0.798	0.735	0.781	0.801

E	K	0.704	0.787	0.826	0.875
	V	0.708	0.756	0.775	0.792
	B	0.762	0.764	0.761	0.784
	D	0.793	0.775	0.802	0.805

K	E	0.724	0.769	0.809	0.822
	V	0.751	0.755	0.817	0.845
	B	0.824	0.765	0.842	0.875
	D	0.817	0.842	0.868	0.869

V	E	0.778	0.836	0.832	0.854
	K	0.813	0.854	0.867	0.889

**Table 6:** F1 value of different methods.

Source	Target	Method			
		SCL-ML	ITIAD	CGRU	Ours
B	D	0.740	0.810	0.812	0.814
	E	0.729	0.794	0.781	0.796
	K	0.782	0.820	0.805	0.835
	V	0.764	0.784	0.797	0.812
	B	0.800	0.820	0.831	0.842

D	E	0.784	0.791	0.812	0.815
	K	0.766	0.798	0.803	0.825
	V	0.821	0.856	0.858	0.921
	B	0.796	0.810	0.812	0.821

E	D	0.803	0.738	0.794	0.824
	K	0.715	0.792	0.830	0.867
	V	0.723	0.761	0.784	0.815
	B	0.786	0.754	0.774	0.790

K	D	0.806	0.764	0.801	0.807
	E	0.735	0.772	0.816	0.821
	V	0.764	0.798	0.824	0.848
	B	0.831	0.787	0.854	0.867
	D	0.829	0.856	0.878	0.879

V	E	0.784	0.843	0.847	0.868
	K	0.838	0.866	0.845	0.870

## Data Availability

The datasets used and/or analyzed during the current study are available from the corresponding author on reasonable request.
